# Nursing experience during COVID-19 pandemic in Korea: a qualitative analysis based on critical components of the professional practice models

**DOI:** 10.1186/s12912-022-01072-0

**Published:** 2022-11-01

**Authors:** Hye-Ryoung Kim, Hwa-Mi Yang

**Affiliations:** 1 College of Nursing, ShinHan University, Dongducheon-si, Republic of Korea; 2 Nursing Department, Daejin University, 1007 Hoguk-ro, 11159 Pocheon-si, Gyeonggi-do Republic of Korea

**Keywords:** Nursing, Professions, COVID-19, Quality improvement, Communication

## Abstract

**Background:**

Nurses have an essential role and responsibility to work at the forefront of patient care during the COVID-19 pandemic. Although the press and public have praised the dedication of nurses in the COVID-19 pandemic, there are several points to consider for nursing professional development. The purpose of this study is to collect the experiences of the nursing profession in the COVID-19 pandemic through interviews, seek improvements for the development of the nursing profession, and suggest directions for the future.

**Method:**

This qualitative study adopts semi-structured interviews analyzing the nursing experience of the COVID-19 pandemic based on the professional practice models (PPMs). Ten nurses with at least two years of working experience and thorough work changes in the COVID-19 pandemic from various settings have participated in the study.

**Results:**

We identified thirty-nine problematic codes and nineteen improvement codes which mapped to 12 key concepts and corresponded to 6 constructs of the PPM model.

**Conclusion:**

Nurses had to take on tasks beyond their duties in urgent situations, which restrained nurses from concentrating on their work. Clarifying working boundaries is fundamental for collaborative care and independent nursing practice. Collaboration and communication among healthcare workers based on mutual understanding can create a respectful working environment. Although there were many difficulties due to the uncertain situation, we can find that the nursing profession can make achievements through systematic and organizational support for sticking to the basics of nursing, securing technical expertise, cultivating critical thinking, and developing various professional attributes. In this way, the establishment of roles based on professional values and duties and the ascertainment of clear boundaries for nursing will ultimately help to improve the quality of patient care.

**Supplementary Information:**

The online version contains supplementary material available at 10.1186/s12912-022-01072-0.

## Background

The World Health Assembly had designated 2020 the International Year of the Nurse and the Midwife [1]. Although it acknowledged nurses’ importance, the nursing community is going through a challenging time in the COVID-19 pandemic.

Nurses have an essential role and responsibility to work at the forefront of patient care during the COVID-19 pandemic and actively participate in the evaluation and monitoring of the community [2]. Some media even portrayed nurses as superheroes because nurses fulfill their duty of care as calling despite the risk of infection. However, In the long-lasting pandemic situation, nurses at the scene of Covid-19 were weighed down by the heavy responsibility and sometimes felt isolated and helpless, overwhelmed by the excessive workload, tired of protective equipment, and feared the uncertainty of infection [3, 4]. Even though nurses take care of patients with a sense of vocation, still, they are treated poorly compared to their duties [5].

Although the press and public have praised the dedication of nurses in the COVID-19 pandemic, there are several points to consider for nursing professional development.

The professional practice models (PPMs) are theoretical frameworks that clarify the nursing role and verify the nursing contribution within the care delivery system [6]. Critical components of PPMs are leadership, nurses’ independent and collaborative practice, environment, nurse development and recognition, research/innovation, and optimal patient outcomes [7]. Analyzing the nursing experience based on critical components of the PPMs will be a meaningful task for advancing nursing.

This study aims to collect the nurses’ experiences in the COVID-19 pandemic, seek improvements for the nursing profession’s development, and suggest future directions.

## Methods

### Study design and participants

This qualitative study adopts semi-structured interviews exploring nurses’ experience in the COVID-19 pandemic. This study followed the “Consolidated criteria for reporting qualitative studies (COREQ)” for methodological integrity [8].

Ten nurses from various settings have participated in the study (Table 1). Inclusion criteria are at least two years of experience, having experience of work changes in the COVID-19 pandemic as a nurse. New nurses or no experience in nursing before the COVID-19 pandemic are excluded.

**Table 1:** General characteristics of the participants

Gender	age	Marital status	Career (years)	Affiliation	Department	Covid19_working place	Covid19_working period
M	27	Single	3	Tertiary University hospital	Trauma intensive care unit	Residential treatment center	1 month
F	42	Married	19	Tertiary University hospital	Integrated nursing care unit	Integrated nursing care unit	18 months
F	29	Single	2.5	Tertiary hospital	Medical intensive care unit	National medical center	5 months
F	28	Single	3	Tertiary hospital	Surgical intensive care unit	Cluster infection epicenter(Daegu-city)	5 months
F	31	Single	5	Tertiary hospital	Medical intensive care unit	Cluster infection epicenter(Daegu-city)	5 months
F	49	Married	19	Public health center	Visiting nurse	Screening center	18 months
F	42	Married	17.2	Tertiary University hospital	OBGY outpatient clinic	Department of infection control of branch hospital	5 months
F	29	Single	3.7	Tertiary University hospital	Intensive care unit of emergency center	National medical center Another care unit	1 month
M	29	Single	4.6	Tertiary University hospital	Trauma intensive care unit	Another care unit	1 month
M	31	Single	5	Secondary hospital	general care unit	COVID-19 speialty care unit	6 months

We recruited participants by purposive and snowball sampling techniques. All participants understood the study purpose and methods and completed informed consent.

### Data collection

Two experienced RN, Ph.D. and University professors conducted in-depth interviews using a semi-structured questionnaire from September 2021 to October 2021.

The semi-structured questionnaires are as follows; (1) What changes have occurred in work (role) in the COVID-19 pandemic? (2) What were the difficulties or problems in the changed situation? (3) What needs to be improved to provide effective nursing care in a situation like this? What support does it need? (4) What have we learned from experiencing COVID-19?

We interviewed in a seminar room with a quiet and comfortable atmosphere and audio recorded. Each face-to-face interview took approximately 45 min to 1 h. During the interview, the researchers took note of essential clues and situations.

We conducted a study process fulfilling Guba and Lincoln’s criteria [9] for the study rigor. For credibility, researchers maintained a value-neutral attitude and collected the expressions of the participants while breaking the prejudice against nurses who performed their work in the COVID-19 pandemic. For dependability, the nurses other than the study participants were asked to read the research results and went through a procedure to verify whether they were meaningful based on their own experiences. We checked whether the collected and analyzed data were consistent with the participant’s experience for confirmability. We complied with inclusion and exclusion criteria for transferability and secured the general characteristics and COVID-19 pandemic working conditions in various nursing environments. Researchers agreed on the data saturation when no more new code appeared.

### Data analysis

Theory-guided thematic analysis was performed based on the PPMs. Prior to the analysis, Researchers defined operationally critical components of the PPMs [7]. Two researchers read the interview scripts repeatedly, coded meaningful units, grouped them according to themes, and then mapped them to theory constructs. After independent analysis, all these processes were conducted through discussions and consensus between researchers.

### Operational definitions for data analysis

- Independent practice: Recognize accountability and ensure autonomy in nurse-led practice and clinical decision-making.
- Collaborative practice: Collaboration with patients and other healthcare workers for quality patient outcomes through effective communication.
- Leadership: Ability to give instructions and directions depending on the context and to practice with professional insights and power.
- Environment: Physical, sociocultural, and organizational factors influencing the quality of nurse-patient relationships and care delivery systems.
- Advancement & recognition: Factors for the development and awareness improvement of the nursing profession, including critical thinking, technical expertise, and support systems.
- Research/Innovation: Methodological issues generating and supporting nursing knowledge and expertise.

## Results

We identified thirty-nine problematic codes and nineteen improvement codes which mapped to 12 key concepts and corresponded to 6 constructs of the PPM model. We presented every code in Table 2 and corresponding statements in the appendix.

**Table 2:** Revealed problems and lessons for improvements from COVID-19

Theoretical constructs	Problematic Codes	Key concepts	Improvement Codes
Independent practice	Passive and dependent tendencies	Autonomy	Nurse-led improvement efforts
	Role Ambiguity and Role Conflict		Working boundaries and delegation for nurses to focus on the nursing work
		Accountability	Nurse accountability
Collaborative practice	Conflict with other health professionals.	Collaboration	Improving the nurse-other healthcare workers, nurse-patient relationship
	Difficulties in nursing due to not cooperative patient		
	Inefficient communication system	Communication	
Leadership	Oppressive leader	Leadership	Decision guide/ control tower/leader
Environment	Resource shortage	Physical setting	Sufficient resource supply
	Inefficient resource allocation		Efficient resource allocation
	Inefficient environmental workflow		
	Inappropriate hospital room assignment		
	Lack of skilled medical staff and resources		The need for strategic standards for the workforce
	Inadequate incident reaction due to working alone		
	Discomfort due to poor environment		
	Difficulty in assessing patient condition		The need for patient monitoring devices
	Unfamiliar work environment	Task complexity & workload	Understanding of work environment
	Difficulties associated with wearing PPE		Enhanced infection control
	Discomfort related to unmet basic physical needs		Application of efficient nursing care delivery system
	Misplacement by a manager with no medical experience		
	The unpredictability of workload		
	Weighted workload		
	Requirement of increased time for the work		
	A culture of not sharing detailed information	Sociocultural environment	Improving public awareness of infection prevention
	Lack of general awareness of the severity		
	Social isolation		
	Sensitive situation	Respectful work environment	
	Complaints (blame) of patient/ patient family		
Advancement & recognition	Misconceptions about nursing professionals	Awareness of nursing professionalism	Improving recognition of nurse professionalism
	The scarlet letters of the confirmed COVID-19 medical staffs		
	Inappropriate response in emergencies	Critical thinking & technical expertise	Enhanced education and training
	Ineffective coping due to inexperienced staffs		
	Lack of work-related training opportunities due to situational difficulties		
	Anxiety/ Fear and powerlessness/ fears of being a transmitter	Support system	Peer support
	The ambivalence between professional duty and individual needs		Encouragement
	Ethical dilemma		The importance of professional supports
	Strain and Psychological conflict concerning not being protected		
	Conflict due to inappropriate pay system		
	Fatigue		
	Lack of support from the Nursing Association		
Research/ Innovation	Ambiguity and vagueness in clinical practice guidelines	Guideline & protocol/Innovation	The need to utilize advanced technology such as artificial intelligence (AI), supporting robots

### Independent practice

The problematic codes revealed in the autonomy, one of the prerequisites for independent practice, were ‘Passive and dependent tendencies’ and ‘Role Ambiguity and Role Conflict.‘

Nurses tended to receive orders and perform tasks passively, finding a straightforward solution in the COVID-19 situation challenging.

*“Nurses are somewhat in a position where they receive orders. So, I think we, nurses, can be passive in some areas, and we tend to be passive when dealing with the overall work in the hospital. Even when it comes to supervisors, we expect them to do something for us by devising a thorough plan instead of doing something on our direct demand. We feel kind of awkward or sorry if we have to make demands on them. We have been so accustomed to being passive, and we tend to think, “Why don’t they give us an answer and wait for it indefinitely.“*

Improvement codes in autonomy were ‘Nurse-led improvement efforts’ and ‘Working boundaries and delegation for nurses to focus on the nursing work.‘

In the COVID-19 situation, nurses experienced the importance of proactive behaviors for optimal patient outcomes. Moreover, these experiences lead to confidence in terms of autonomy.

*“As our role and importance grew, I think it gave us an opportunity to have a voice. That’s why we need something like this in reverse. We’ve been doing things like this little by little… Would you say that something that was passive compared to the past has become more active? As the nurses talked more proactively, the solution came out sooner (laughs).“*

Because of the coordinator role of nurses, working boundaries are often obscure. However, clarifying working boundaries so that nurses can focus on their work is a top priority for the autonomy of the nursing profession.

*“One of the things that I requested the most was to exclude tasks like visitor control that anyone can do, even not a nurse. But first, all things related to the ward are transferred to the nursing field. When we fight such a difficult battle, it’s a little bit… Being able to entrust that part in an area where such nursing doesn’t have to be directly involved, I think that’s the top priority.“*

In COVID-19, public expectations of nurses affect their professional accountability, and this perception serves as a development factor for independent nursing practice.

*“Nurses are the ones who run the most, and the media is talking about them with a lot of emphases now, and the atmosphere is so good that nurses really need nurses in such a difficult situation; they are helping me a lot, and taking care of their health It must be difficult, but anyway, it is a job, but I have seen many things like that, taking responsibility in a difficult environment where you have to go out and work, and the patients. And it seems that our nurses themselves recognize that they are playing a very important.“*

### Collaborative practice

In collaboration, an axis of collaborative care, problematic codes were ‘Conflict with other health professions’ and ‘Difficulties in nursing due to not cooperative patient.‘

In responding to uncooperative patients, nurses appear exhausted, and conflicts with other healthcare workers are likely to occur in a tense situation.

*“There are cases where we get into subtle conflicts with other professions. We are working on it as fast as possible, but it is not very pleasant for doctors if it does not work out. So, they end up yelling at us.“*

Another axis of collaborative care in communication, ‘Inefficient communication system,‘ was the problem.

*“Usually, when you work as a nurse in a hospital, you communicate directly with a doctor. However, the communication process is more complicated for a dispatched nurse.“*

### Leadership

The oppressive leader adversely affects autonomy, leads to work inefficacy, and the leader who gives an appropriate decision guide is critical.

*“We need someone at the center giving directions. It does not matter who it is. Whether that person is a nurse, a professor, or whoever it is, there were cases where such people gave instructions from the central no matter how urgent it was. Things can end a little faster if things come and go in order.“*

### Environment

The environment was categorized into physical setting, task complexity & workload, sociocultural and respectful environment.

Nurses had increased task complexity & workload in the covid19 situation. Related codes include an unfamiliar work environment, difficulties associated with wearing PPE, discomfort related to unmet basic physical needs, misplacement by a manager with no medical experience, the unpredictability of workload, weighted workload, and a requirement of increased time for the work. Especially, nurses dispatched to the COVID-19 outbreak had faced difficulties as they undertook work in an unfamiliar environment.

*“First of all, we cannot guide people with everything. After being in the ICU, I did not fully understand how things worked there when I went to the general ward. Working at an unfamiliar hospital was challenging.“*

The difficulties associated with wearing PPE were also factors that added to the workload.

Moreover, The deployment of duties by public officers with no healthcare experience made nurses spend much time and energy adapting to the work, reducing work efficiency and, finally, patient outcomes.

*“Public officials with no medical experience are usually in charge of deployment. They often fail to consider the importance of a nurse’s career break or the specific department career. From my experience, I don’t think they are dispatching the right experienced staff to the right place.“*

The lesson for improving nursing practice in task complexity and workload categories were ‘needs for an orientation to the work environment,‘ ‘strengthening infection control at the organizational level,‘ and ‘applying an efficient nursing delivery system.‘

Concerns about public criticism of the COVID-19 outbreak in hospitals kept hospitals from sharing coping information.

*“It was hard to make something formal and share it among the nurses because it did not seem pleasant to have it exposed to the media at the beginning. The hospital tried to get things done quietly and did not want the information to be public, so details were not shared. Those were some of the frustrating and challenging parts.“*

In addition, the lack of public awareness of the seriousness of COVID-19 has aggravated the difficulty of infection control, and social isolation due to the possibility of infection transmission made it even more difficult for nurses.

Sensitive situations from COVID-19 and complaints from patients or their families made an exclusive environment, far from a respectful environment, inhibiting interdisciplinary collaboration as the basis for quality care.

### Advancement & recognition

The key concepts found in Advancement & Recognition were awareness of nursing professionalism, critical thinking & technical expertise, and support system.

Misunderstandings about the nursing profession and social stigma such as scarlet letters on the medical staff with COVID-19 made it difficult for nurses.

*“Nurses have tasks quite different from doctors. However, due to the nature of the hierarchical system in Korea, many patients think that nurses can only do something under the doctor’s instructions. As a result, most patients and guardians often ignore nurses unconsciously, which seems to cause many problems.“*

Improvement of recognition of the nursing profession should be the priority.

*“Oh, but actually, the atmosphere has changed a little after going through this period. I think that the people’s awareness that nurses are really working hard and needed in hospitals has become stronger.“*

Ineffective coping of inexperienced nurses and lack of training opportunities were negative codes of critical thinking & technical expertise. It is necessary to improve this through enhancing education and training.

There were concerns about how to care for clients because of the uncertainty of the COVID-19 pandemic. Still, in the end, fundamental nursing skills such as strengthening infection control, respiratory care, and emotional support are essential.

*“Since we are dealing with confirmed patients, we have to pay more attention to sterilization. Infection control has become more critical, such as donning protective equipment.“*

Various negative psychological responses, the ambivalence between professional duty and individual needs, ethical dilemmas, strains, conflicts, and lack of organizational support revealed problems in the support system. Peer support, encouragement, and professional support systems were improvement codes.

*“When we were working, a professor at a tertiary hospital in the metropolitan area once or twice a week came to us and gave us advice, and there were many things like doing procedures that couldn’t be done. However, it was a pity that there was not enough time for staff training, but anyway, it seemed like a ray of light. Every time the professor comes, doctors and nurses also became more anticipating that time.“*

### Research/Innovation

Ambiguity and vagueness in clinical practice guidelines were problematic codes in guidelines & protocol.

*“I hope that something in the protocol can respond quickly according to this time. But now, it was challenging and complex for everyone to respond from time to time. It was different depending on the local government. Each hospital had a different level or degree of thinking about it, so there was no clear guideline.“*

The need to utilize advanced technology such as artificial intelligence (AI) and supporting robots was improvement code in Innovation.

*“Seriously, nurses have a lot of repetitive words like machines. But now, for example, if we replace them with kiosks or artificial intelligence or medical support robots, they say the same thing over and over again, and I think these repetitive can be improved a lot.“*

## Discussion

There were several structural and technical problems in the COVID-19 pandemic, as shown in the results, but the nurses tried to find a solution with the best efforts in their situation. The COVID-19 pandemic has been a harsh situation for nurses, but it has also been an opportunity to feel confident and leap forward.

In the COVID-19 situation, nurses experienced the importance of proactive behaviors based on critical thinking and technical expertise for optimal patient outcomes in collaboration with other healthcare personnel. Moreover, these experiences lead to confidence in terms of autonomy. Clarifying working boundaries so that nurses can focus on their work is a top priority for the autonomy of the nursing profession. In COVID-19, public expectations of nurses affect their professional accountability, and this perception serves as a development factor for independent nursing practice.

Fostering critical thinking and improving technical expertise through enhancing education or training affects the independence of nursing through autonomy, evidenced by active participation in decision-making [10]. A study that suggested the directions of nursing education the post-COVID-19 also insisted that it is crucial to provide opportunities to learn critical thinking and flexibility in a changing healthcare environment [11]. Solution-based education, which can provide an opportunity to properly enhance basic nursing skills and coping abilities to respond to various clinical situations, may foster critical thinking and technical expertise [12]. Besides, It is necessary to seek alternative ways to teach, such as virtual learning in difficult educational situations, the COVID-19 pandemic [13].

The independence of nursing practice presupposes an apparent working boundary establishment [14, 15]. Interprofessional congruent working boundaries through mutual understanding and cooperation are the basis for quality patient outcomes [12, 16]. As coordinators, nurses participate in various patient-related tasks [17]. Even though the coordinator’s role does not mean to perform the task directly, there were many cases in which nurses had to take on tasks that went beyond their duties in urgent situations, restraining them from concentrating on their work. It is necessary to rationally re-establish nurses’ role as coordinators applicable to practice.

In addition, it is necessary to promote collaborative care by developing an interprofessional education program, providing an opportunity for mutual understanding and cooperation among healthcare workers [18, 19].

Multi-dimensional environmental support focusing on nursing is also required [20, 21]. Significantly, a respectful working environment affects quality communication and contributes to successful collaborative practice [21].

Nurses have the right to work in a safe environment to express themselves without hesitation [22]. It cannot sustain quality patient outcomes based on the excessive sacrifice of nurses. Basic physiological needs, such as rest, food, and sleep, must be advocated. Utilizing advanced technology such as artificial intelligence and supporting robots might be helpful. To this end, infrastructure and institutional process reforms are required [22].

The role of associations in professional development is also crucial [23]. Efforts at the association level to establish a policy on adequate nursing staff or a sound payment system and a systematic and organizational support system are essential elements in developing the nursing profession [24].

### Implications for policy, practice, and research

In the future, developing solution-based education content for cultivating critical thinking and technical expertise and researching various effective education delivery methods is necessary.

Efforts are needed to develop educational programs and provide opportunities for mutual understanding and cooperation among healthcare workers.

We propose research to study the role or contribution of nursing managers based on the PPMs and develop guidelines or protocols that appropriately respond to challenging and complex situations.

### Limitations of the study

We presented the directions for improving nursing professions and quality nursing. Nevertheless, this study has some limitations. This study is qualitative, and the number of participants is relatively small. In addition, since conducting with nurses in Korea, there may be generalization issues in countries with different institutional and cultural support.

## Conclusion

Nurses had to take on tasks beyond their duties in urgent situations, which restrained nurses from concentrating on their work. Clarifying working boundaries is fundamental for collaborative care and independent nursing practice. Collaboration and communication among healthcare workers based on mutual understanding can create a respectful working environment. Although there were many difficulties due to the uncertain situation, we can find that the nursing profession can make achievements through systematic and organizational support for sticking to the basics of nursing, securing technical expertise, cultivating critical thinking, and developing various professional attributes. In this way, the establishment of roles based on professional values ​​and duties and the ascertainment of clear boundaries for nursing will ultimately help to improve the quality of patient care.

## Electronic supplementary material

Below is the link to the electronic supplementary material.

Supplementary Material 1

## Data Availability

The data supporting this study’s findings are available from the corresponding author, [H-M Yang], upon reasonable request.
