# Supplementary material for: Nursing experience during COVID-19 pandemic in Korea: a qualitative analysis based on critical components of the professional practice models
Source: BMC Nurs. 2022 Nov 1;21:288. doi: 10.1186/s12912-022-01072-0 (PMC9623934; doi:10.1186/s12912-022-01072-0)
Supplement: Supplementary file 1 — Supplementary Material 1 [file 12912_2022_1072_MOESM1_ESM.docx]

| Appendix 1. Problems revealed in the COVID-19 situation | | | |
| --- | --- | --- | --- |
| Key concepts | category | Problematic code | statements |
| Independent practice | Autonomy | Passive and dependent tendencies | Nurses are somewhat in a position where they receive orders. So, I think we, nurses, can be passive in some areas, and we tend to be passive when dealing with the overall work in the hospital. Even when it comes to supervisors, we expect them to do something for us by devising a thorough plan instead of doing something on our direct demand. We feel kind of awkward or sorry if we have to make demands to them. We have been so accustomed to being passive, and we tend to think, "Why don't they give us an answer and wait for it indefinitely." |
|  |  | Role Ambiguity and Role Conflict | It usually takes at least 10 to 15 minutes to don and doff the protective equipment. As a result, doctors and other professions were very reluctant to come in. They thought it was a waste of time. So, they often did not want to come in even when needed. Consequently, nurses often had to work on tasks, which were not part of our job description. |
| Collaborative practice | Collaboration | Conflict with other health professionals | There are cases where we get into subtle conflicts with other professions. We are working on it as fast as possible, but it's annoying for doctors if it does not work out. So, they end up yelling at us. |
|  |  | Difficulties in nursing due to not cooperative patient | The patient looked for his items and checked cash in his wallet in case anyone stole it. He found his cell phone and shouted, "I should go to the bathroom right now." The low oxygen saturation level continued to get lower due to the patient's agitation and screaming. Three or four nurses rushed to the room to calm the patient, but it was not enough. We asked for cooperation from the patient by reminding him of his current physical condition and a hospital. Still, he didn't listen to us at all, and he continued to swear at us. He expressed his anger by saying, "I am a patient. So, you need to do what I say." I was stunned when I heard that. Then I started to wonder, "I am a nurse. I am a medical staff, but why don't you cooperate in our hospital? I didn't do anything wrong, but why should I get cursed at all?". |
|  | Communication | Inefficient communication system | Usually, when you work as a nurse in a hospital, you communicate directly with a doctor. However, when you are a dispatched nurse, the communication process is a lot more complicated. |
| Leadership | Leadership | Oppressive leader | When nurses get dispatched to a different hospital or ward, it is not usually out of their own will. Instead, they mostly followed the instruction of the head nurse. |
| Environment | Physical setting | Resource shortage | Even if we work without a break, we feel that we need more workforce and don't have time to provide sufficient care to patients. |
|  |  |  | It would be nice if there were sufficient medical supplies in stock. However, we had to request more or use the same items again after cleaning it, even though it is not advisable. |
|  |  | Inefficient resource allocation | We often had problems due to the inappropriate placement of staffing. One nurse in the intensive care unit (ICU) takes care of two to four patients on average. Nurses in the general ward take care of 12-24 patients on average, and five to ten in the case of a comprehensive ward. As such, patients in the ICU need intensive observation. We have to react more sensitively or faster than the patients in the general ward. On the contrary, nurses in ICU are less flexible. They have more difficulty building rapport with patients than those in the other wards. |
|  |  | Inefficient environmental workflow | I have been to the general ward a day for support. However, the supplies are often only located in a nurse station, not in a patient room. I wore Level D for a moment, but I had to doff and return if I left anything behind in a station. It was quite a burden. |
|  |  | Inappropriate hospital room assignment | There were different kinds of patients regardless of severity: trauma patients, patients who could walk freely, and even babies. Indeed, a baby came in with his family member even if he was not a confirmed COVID-19. |
|  |  | Lack of skilled medical staff and resources | These difficulties do not only come from a lack of nurses or doctors. It is also due to a lack of beds, various machines, and skilled personnel. |
|  |  | Inadequate incident reaction due to working alone | I took charge of one floor while wearing Level D and usually came out after an hour or two. Since we go in one by one, if patients start acting out, there are difficulties. |
|  |  | Discomfort due to poor environment | It was a little cold when we went back and forth from outside. When I work indoors, it's hot because of the poor ventilation. |
|  |  | Difficulty in assessing patient condition | If we watch the patients closely by having them next to us, we can manage the changes right away. However, in residential treatment centers, we only talk to patients on the phone and rarely meet them in person. As a result, the medical staff could not respond quickly to the patient's situation to undergo emergency intubation. |
|  | Task complexity & workload | Unfamiliar work environment | First of all, we cannot guide people with everything. After being in the ICU, I did not fully understand how things worked there when I went to the general ward. Working at an unfamiliar hospital was challenging. I didn't know the ins and outs of the hospital and the staff there. It was more challenging since I wasn't the staff who had worked there for a long time. |
|  |  | Difficulties associated with wearing PPE | The hardest part was wearing a gown and level D, and it was hard to work in PPE, as it requires lots of energy. I lost time or accuracy due to dull senses when I obtained IV access while wearing two sets of gloves. Wearing protective glasses gives blurry vision to a person with glasses like me. They work in a powered air-purifying respirator (PAPR) itself is problematic in communication because of the sound of the wind inside the hood. |
|  |  | Discomfort related to unmet basic physical needs | I didn't even go to the bathroom because I didn't know much about COVID-19, and I thought I had to endure it once I don PPE. So, I didn't even drink much water. |
|  |  | Misplacement by a manager with no medical experience | Public officials with no medical experience are usually in charge of deployment. They often fail to consider the importance of a nurse's career break or the specific department career. From my experience, I don't think they are dispatching the right experienced staff to the right place. |
|  |  | The unpredictability of workload | The number or severity of COVID-19 confirmed patients seems to be more unpredictable. So, when you feel like you require more staffing, work gets more challenging. Among the additionally deployed nurses, there was a nurse suspected of contacting a confirmed patient. All the nurses had to leave. Only the existing nurses were left in the hospital to take care of the patients, so I remember it being one of the most challenging times. |
|  |  | Weighted workload | Initially, patients are not allowed to take their belongings into the ICU. However, we often have them stored in our workstations because they belong to patients with infectious diseases. As the number of patients increased, there were many times where we had to contact their guardians. Still, it was difficult to get them immediately when something happened. Furthermore, they often forcefully demanded that we find the patient's belongings. |
|  |  | Requirement of increased time for the work | Since COVID-19 is a respiratory disease, there's a situation where the saturation drops, and we have to conduct intubation urgently. Considering how pressing the case is, we had to do it as soon as possible. However, it takes too much time preparing, such as wearing level D (protective clothing) and carrying lots of plastic bags. So we cannot handle the situation quickly, even in emergencies. |
|  | Sociocultural environment | A culture of not sharing detailed information | It was hard to make something formal and share it among the nurses because it did not seem pleasant to have it exposed to the media at the beginning. The hospital tried to get things done quietly and did not want the information to be public, so details were not shared. Those were some of the frustrating and challenging parts. |
|  |  | Lack of general awareness of the severity | I don't think those people who are not in the medical field took the situation seriously. They did not seem to understand its seriousness, and they just thought, "as long as I don't get Covid-19, then it's fine." I believe this lack of awareness of the seriousness of COVID-19 helped spread the disease. |
|  |  | Social isolation | Some mothers of schoolchildren or kindergarteners were reluctant to allow their kids to contact my children as I work in a hospital. Even some mothers were unwilling to meet me in person as I was a nurse. |
|  | Respectful work environment | Sensitive situation | Patients are currently being quarantined in a narrow space. Since we are in a sensitive situation, we are more cautious with what we say. |
|  |  | Complaints (blame) of patient/ patient family | There are now more complaints, such as patients complaining about social distancing not being observed while waiting or complaining about other patients not wearing masks. |
|  |  |  | Actually, a lot of guardians complained. How did you treat the patient so that it got worse? |
| Advancement & recognition | Awareness of nursing professionalism | Misconceptions about nursing professionals | Nurses have tasks quite different from doctors. However, due to the nature of the hierarchical system in Korea, many patients think that nurses can only do something under the doctor's instructions. As a result, most patients and guardians often ignore nurses unconsciously, which seems to cause many problems. A patient is a person who needs care and is hospitalized for treatment. However, I hope they do not build the wrong rapport with us, thinking that "nurses need to do whatever I say since I am a patient." |
|  |  | The scarlet letters of the confirmed COVID-19 medical staffs | In fact, all the nurses were angry. There is a possibility for those working at the hospital to get infected, but we all thought that I did not want to be the first one to be infected. However, among the staff in the hospital, a nurse became the first infected person. Everyone was very heartbroken. The announcement, however, was made to the entire staff. The nurse was a new hire, and the exposure to the whole members must have been difficult for her. |
|  | Critical thinking & technical expertise | Inappropriate response in emergencies | We go into the room without proper protective clothing in an emergency, even if the COVID-19 test results have not come out yet. We knew that it would be risky to go in without knowing the test results, but what can we do? We have to go in if it's an emergency, no matter what. I don't care about myself... I only wear a mask without proper PPE and go in... These risky situations occur all the time. |
|  |  | Ineffective coping due to inexperienced staffs | Honestly, it must have been the first time for staff to deal with COVID-19 cases and never simulated before, and its severity was so high. So, effective coping was difficult without experience. |
|  |  | Lack of work-related training opportunities due to situational difficulties | It was not education. It was just a notice asking us to do this and that. However, all we could do then was to follow the quarantine rules. That was why we kept emphasizing it and did nothing else. Fewer education opportunities were also a big problem. |
|  | Support system | Anxiety/ Fear and powerlessness/ fears of being a transmitter | I began to be truly scared, watching the rapid progress of the disease for patients who were getting better. I have seen dear patients die shortly after tracheal intubation. Because of these repetitive experiences, the moment another patient gets intubated makes me tear up thinking if I should prepare farewell with him. |
|  |  |  | I heard that the nurses were reluctant to go home even if they were off duty for quite a while. They were not afraid of being infected; instead, they stayed in the hospital to not become some infection source. |
|  |  | The ambivalence between professional duty and individual needs | There were nurses who were worried that they might get sick, and nurses worried that their coworkers might get sick. I think these thoughts coexisted. When I get sick, I feel sorry for my coworkers because I knew they had to work harder to make up for my absence. However, it was also true that I wanted to take a rest by getting sick. These were some mixed thoughts. |
|  |  | Ethical dilemma | Should I say it was a conflict of ethics? Those conflicts gave me a hard time psychologically. As nurses, we feel proud of our job when we take care of patients and see improvements in their conditions thanks to our proper care. However, with COVID-19 cases, our care does not necessarily improve patients' conditions. A sudden case of hypoxia happened to patients because of the miscalibrated amount of sedatives or too much injected. I applied for the COVID-19 ward to find a sense of duty as a nurse, but I ended up feeling the "Corona Blue." |
|  |  | Strain and Psychological conflict concerning not being protected | It is not fair to do other professionals' jobs. However, We should do it and deal with it in case of medical accidents resulting from it. As dispatched and not affiliated nurses, our voices were not heard even if we told them about the difficulties, made petitions, or wrote a report. That's why I had a hard time psychologically. I was doing the tasks for which I wondered if it was part of my job, and I was nervous, but most of all, I felt like I was at risk. |
|  |  | Conflict due to inappropriate pay system | Those who came back after a break gets paid 6 to 7 million won a month. However, most nurses from national medical centers get the same salaries as before with a higher level of work intensity, so they often get tired of it and quit. |
|  |  | Fatigue | After all these, I feel exhausted. These days, when the temperature is high, it is so hot to a point where I feel like collapsing on the spot. |
|  |  | Lack of support from the Nursing Association | Other occupational associations enlist people from all over the country and guide them to make applications. Still, the nursing association is not doing enough, even though they are trying. |
| Research/  Innovation | Guideline & protocol | Ambiguity and vagueness in clinical practice guidelines | I hope that there is something in the protocol that can respond quickly according to this time. But now, it was challenging and complex for everyone to respond from time to time. It was different depending on the local government. Each hospital had a different level or degree of thinking about it, so there was no clear guideline. |

| Appendix 2. Lessons for improvements from COVID-19 | | | |
| --- | --- | --- | --- |
| Key concepts | category | Improvement code | statements |
| Independent practice | Autonomy | Nurse-led improvement efforts | As our role and importance grew, I think it gave us an opportunity to have a voice. That's why we need something like this in reverse. We've been doing things like this little by little... Would you say that something that was passive compared to the past has become more active? As the nurses talked more proactively, the solution came out sooner (laughs). |
|  |  | Working boundaries and delegation for nurses to focus on the nursing work | One of the things that I requested the most was to exclude tasks like visitor control that anyone can do, even not a nurse. But first, all things related to the ward are transferred to the nursing field. When we fight such a difficult battle, it's a little bit... Being able to entrust that part in an area where such nursing doesn't have to be directly involved, I think that's the top priority. |
|  | Accountability | Nurse accountability | Nurses are the ones who run the most, and the media is talking about them with a lot of emphases now, and the atmosphere is so good that nurses really need nurses in such a difficult situation, they are helping me a lot, and taking care of their own health It must be difficult, but anyway, it's a job, but I've seen a lot of things like that, taking responsibility in a difficult environment where you have to go out and work, and the patients. And it seems that our nurses themselves recognize that they are playing a very important |
| Collaborative practice | Collaboration | Improving the nurse-other healthcare workers, nurse‐patient relationship | A nurse's competency is essential for practical nursing, but it also requires other healthcare workers' and patients' cooperation. |
| Leadership | Leadership | Decision guide/ control tower/leader | We need someone at the center giving directions. It doesn't matter who it is. Whether that person is a nurse, a professor, or whoever it is, there were cases where such people gave instructions from the central no matter how urgent it was. If things come and go in order, things can end a little faster |
| Environment | Physical setting | Sufficient resource supply | A lot of treatment materials are needed. I think sufficient supplies and supports make possible efficient care. |
|  |  | Efficient resource allocation | I think the proper arrangement of nurses and medical staff is necessary, such as nurses from ICU to ICU and from the general ward to the wards. In addition, it will be possible to increase staffing to a certain extent by appropriately adjusting the days off for dispatched nurses. But above all else, proper placement is essential. |
|  |  | The need for strategic standards for the workforce | Since the number of nursing personnel is not accurately specified by law, it seems necessary to establish appropriate nursing personnel standards. |
|  |  | The need for patient monitoring devices | Well, First of all, I have to keep watching, so, for example, I think it should always be CCTV.  I think the patient should always be seen.  I am not sure what the isolated patient's condition is. I can't even make calls... So I think it would be very helpful to have a CCTV. |
|  | Task complexity & workload | Understanding of work environment | If we unfamiliar with the department, it would be helpful to orient and guide us about the location of items and the protocol within the department before works. |
|  |  | Enhanced infection control | Since we are dealing with confirmed patients, we have to pay more attention to sterilization. Infection control, such as donning protective equipment, has become more critical. |
|  |  | Application of efficient nursing care delivery system | In team nursing, only the patient in charge is identified and handed over, so team nursing can shorten the handover time or subdivide the division of work, so it would be better to proceed in this direction. |
|  | Sociocultural environment | Improving public awareness of infection prevention | It made me think a lot that this is not the end just because we care. The most necessary thing for us to deal with wisely is to reduce the number of confirmed cases. I thought a lot that it would be good if everyone paid a little more attention to quarantine. |
| Advancement & recognition | Awareness of nursing professionalism | Improving recognition of nurse professionalism | Oh, but actually, after going through this period, the atmosphere has changed a little. I think that the people's awareness that nurses are really working hard and that nurses are really needed in hospitals has become stronger. |
|  | Critical thinking & technical expertise | Enhanced education and training | I thought emergency education is necessary very well, and at least one or two more skilled doctors or skilled nurses should be more appropriate. |
|  | Support system | Peer support | As a nurse, if you get sick, you feel sorry for your colleagues. But it's true that if you get sick again, you want to rest. There were subtle things like that, but I was aware that it was unavoidable because I was in pain, and I think other co-workers were willing to go to work and make a lot of effort in that regard. |
|  |  | Encouragement | Ummm. First of all, it gives me strength to just say thank you. While saying thank you to the COVID-19 nurses and sending flowers, of course, I don't want these things, but hearing these words gave me strength. |
|  |  | The importance of professional supports | When we were working, a professor at a tertiary hospital in the metropolitan area once or twice a week came to us and gave us advice, and there were many things like doing procedures that couldn't be done. However, it was a pity that there was not enough time for staff training, but anyway, it seemed like a ray of light. Every time the professor comes doctors and nurses also became more anticipating that time. |
| Research/ Innovation | Innovation | The need to utilize advanced technology such as artificial intelligence (AI), supporting robots | Seriously, nurses have a lot of repetitive words like machines. But now, for example, if we replace them with kiosks or artificial intelligence or medical support robots, they say the same thing over and over again, and I think these repetitive can be improved a lot. |
